# The comorbidity between migraine and hypothyroidism

**DOI:** 10.1186/1129-2377-14-S1-P138

**Published:** 2013-02-21

**Authors:** C Lisotto, F Mainardi, F Maggioni, G Zanchin

**Affiliations:** 1 Headache Centre, University of Padua, Italy; 2 Headache Centre, Hospital of Venice, Italy

## Introduction

Comorbidity refers to greater than coincidental association of separate conditions. The International Headache Society classification brought as new entry the headache attributed to hypothyroidism. The diagnostic criteria require the headache to resolve within 2 months after effective treatment of hypothyroidism (HT). This condition seems to be rare in clinical practice, whereas it is more common to see migraine patients also affected by HT.

## Objectives

To demonstrate the comorbidity between migraine and HT.

## Materials and methods

We retrospectively evaluated the clinical records of 3,727 patients diagnosed with primary headaches referred to our Headache Centre from 2005 to 2011.

## Results

The population included 2,232 patients with migraine without aura (MO), 485 with tension-type headache (TTH), 367 with MO + TTH, 228 with migraine with aura (MA), 203 with MO + MA, 143 with cluster headache, and 69 with other primary headaches. Overall, 98 cases (95 females and 3 males) with full-blown HT requiring hormone therapy were noted. Ninety of these cases (2 males) were migraineurs and 8 suffered from TTH. Therefore, the prevalence of HT was 3.0% in migraine and 1.6% in TTH. Interestingly, HT occurred after migraine onset in 87 patients (96.7%), whereas it preceded migraine in 2 MO subjects and in 3 TTH patients. In a population-based study the prevalence of HT resulted to be 0.84% [1]. In our clinic-based survey the prevalence of HT in migraineurs was 3.0%. For 52.0% of patients the headache showed a significant worsening after the onset of HT and hormonal replacement therapy. It is challenging to speculate whether the worsening could be attributable to the hormonal disorder, to levothyroxine treatment or both.

## Conclusion

We found a high prevalence of HT in migraine, significantly higher than in the general population. HT should be considered as one of the variety of migraine comorbidities, even if possible pathophysiologic relationships remain unclear. This is the first study that reports the comorbidity between the two conditions.
